# Health-related quality of life one year after the diagnosis of oesophageal cancer: a population-based study from the Swedish National Registry for Oesophageal and Gastric Cancer

**DOI:** 10.1186/s12885-021-09007-9

**Published:** 2021-11-26

**Authors:** Berit Sunde, Mats Lindblad, Marlene Malmström, Jakob Hedberg, Pernilla Lagergren, Magnus Nilsson

**Affiliations:** 1 Division of Surgery, Department of Clinical Science Intervention and Technology, Karolinska Institutet and Department of Upper Abdominal Diseases, Karolinska University Hospital, SE-14186 Stockholm, Sweden; 2 Lund University, Department of Health Sciences and Department of surgery, Skane University Hospital, 221 85 Lund, Sweden; 3 Department of Surgical Sciences, Uppsala University, 751 85 Uppsala, Sweden; 4 Surgical Care Science, Department of Molecular Medicine and Surgery, Karolinska Institutet, Karolinska University Hospital, 171 76 Stockholm, Sweden; 5 Department of Surgery and Cancer, Imperial College London, London, SW7 2AZ UK

**Keywords:** PROM, HRQOL, Oesophageal Cancer, Epidemiology, Palliative, Surgery and Chemoradiotherapy

## Abstract

**Background:**

Population-based patient reported outcome data in oesophageal cancer are rare. The main purpose of this study was to describe health-related quality of life (HRQOL) 1 year after the diagnosis of oesophageal cancer, comparing subgroups of curatively and palliatively managed patients.

**Methods:**

This is a nationwide population-based cohort study, based on the Swedish National Registry for Oesophageal and Gastric Cancer (NREV) with prospectively registered data, including HRQOL instruments from the European Organisation for Research and Treatment of Cancer including the core and disease specific questionnaires (EORTC QLQ-C30 and QLQ-OG25). Patients diagnosed with oesophageal cancer between 2009 and 2016 and with complete HRQOL data at 1 year follow-up were included. HRQOL of included patients was compared to a reference population matched by age and gender to to a previous cohort of unselected Swedish oesophageal cancer patients. Linear regression was performed to calculate mean scores with 95% confidence intervals (CI) and adjusted linear regression analysis was used to calculate mean score differences (MD) with 95% CI.

**Results:**

A total of 1156 patients were included. Functions and global health/quality of life were lower in both the curative and palliative cohorts compared to the reference population. Both curatively and palliatively managed patients reported a severe symptom burden compared to the reference population. Patients who underwent surgery reported more problems with diarrhoea compared to those treated with definitive chemoradiotherapy (dCRT) (MD -14; 95% CI − 20 to − 8). Dysphagia was more common in patiens treated with dCRT compared to surgically treated patients (MD 11; 95% CI 4 to 18). Those with palliative intent due to advanced tumour stage reported more problems with dysphagia compared to those with palliative intent due to frailty (MD -18; 95% CI − 33 to − 3).

**Conclusions:**

One year after diagnosis both curative and palliative intent patients reported low function scores and severe symptoms. Dysphagia, choking, and other eating related problems were more pronounced in palliatively managed patients and in the curative intent patients treated with dCRT.

## Introduction

Currently oesophageal cancer is the seventh most common cancer and the sixth most common cause of cancer deaths worldwide [1]. Oesophageal cancer is a lethal disease, with approximately 70% of patients being managed with a palliative intention after initial staging. More than half of the patients initially managed curatively, eventually also succumb to the disease [2]. Despite that the majority of patients diagnosed with oesophageal or gastro-oesophageal junctional (GOJ) carcinoma are managed with palliative intent, most of the data published regarding health-related quality of life (HRQOL) addresses curative treatment [3].

The importance of symptom management and HRQOL after cancer diagnosis has recently gained more attention in clinical cancer research [4, 5]. In order to understand and better meet the needs of the patients, we continuously need knowledge of patients’ experiences of their disease in all the different stages and treatment situations. In ongoing and future trials HRQOL is an important outcome, ideally measured before, during and after treatments and also in the evaluation of different clinical management pathways [3]. Reporting of HRQOL outcomes in palliative patients has recently increased in high incidence diseases such as breast, colorectal, prostate, and lung cancer, while less data is to date available from patients with oesophageal cancer [6, 7].

HRQOL after curative surgical intent treatment of oesophageal cancer has been described extensively, based on data from several trials and cohort studies [8–12], although to our knowledge, there are no published data directly comparing patients operated with those who are treated with curatively intended, so-called definitive chemoradiotherapy (dCRT), without surgery. As mentioned above, data on patient reported HRQOL in patients managed palliatively is scarce, and there is no documented knowledge regarding HRQOL in palliative subgroups, for example by the reason for the palliative management intention or type of therapy. Published studies on palliative patients have mainly focused on dysphagia symptom management [7, 13–15] and there is a clear knowledge gap regarding the overall HRQOL in unselected patients with oeophageal cancer [6, 16].

The main aim of this study was to describe patient reported HRQOL 1 year after the diagnosis of oesophageal and GOJ carcinoma in a nation-wide population-based cohort, in unselected patients with all tumour stages, levels of performance status and comorbidity, following the various forms of palliative and curatively intended management. A further aim was to analyse HRQOL in curative intent subgroups by T-stage and treatment type and in palliative intent subgroups by the reason for palliative management, i.e. distant metastatic disease, locally irresectable tumour, or other reasons for palliative treatment intent.

## Methods

### Study design

This cohort study, aimed to map the HRQOL landscape in an unselected national cohort of oesophageal or GOJ Siewert type I and II cancers, comprising both patients with curative and palliative treatment intention. A study cohort was defined with data collection between January1 ^st^, 2009, and December 31st, 2017, using prospectively registered exposure and outcome data from the Swedish National Registry for Esophageal and Gastric Cancer (NREV). All patients diagnosed with oesophageal or GOJ Siewert types I and II cancers and alive 1 year after diagnosis, were included in the study cohort.

### The Swedish National Registry for Oesophageal and gastric Cancer (NREV)

NREV is a Swedish national registry, launched in 2006, collecting information on all patients diagnosed with oesophageal or gastric cancer in Sweden. The data collection includes information regarding staging, performance status, comorbidity, multidisciplinary team conference recommendation of treatment intention and actual treatments administered.

In addition, questionnaires regarding PROM (patient reported outcome measures) are sent to all patients alive 1 year after diagnosis. To this end, the European Organisation for Research and Treatment of Cancer’s (EORTC) written HRQOL assessment questionnaire QLQ-C30 [17] and the oesophageal symptom specific questionnaire module QLQ-OG25 [18] are used.

Since data collection started on January 1 ^st^, 2006 and until this data extraction in May 2018, approximately 7800 oesophageal and GOJ cancers have been registered. The registry has been validated and is considered to have a good coverage of 95% of these cancers diagnosed in Sweden, and to contain highly valid data [19] . Data are continuously monitored and followed- up by the six regional cancer centres in Sweden.

#### Patient subgroups under study

In the registry, the treatment intention after diagnosis and staging was entered as either curative or palliative, usually following a consensus decision at a multi-disciplinary team conference (MDT) shortly after diagnosis and completion of the initial staging of the cancer. Within the curative intent group, patients were subclassified by T-stage to either a T0-T1 group, mainly managed endoscopically and a T2-T4 group, which was further subclassified by treatment with oesophagectomy (with or without neoadjuvant therapy) or dCRT, in which patients were treated curatively without surgery. The patients that after diagnosis and staging were classified as managed with palliative intention upfront, and still alive at 1 year after diagnosis and with completed HRQOL questionnaires, were subdivided into three groups by the reason for the palliative treatment intention: distant metastatic disease (M1), locally irresectable tumour (T4b), and non-tumour related reasons, mainly frailty with predicted inability to tolerate the demanding curative therapy. In a sensitivity analysis, a comparison between responders and non-responders to HRQOL questionnaires was performed with description of differences regarding the American Society of Anesthesiologists (ASA) comorbidity score, age, gender, performance status (WHO), histological subtype and clinical stage.

#### Outcomes

The primary outcome of the study was patient reported HRQOL scores 1 year after cancer diagnosis. In order to facilitate interpretation, the HRQOL scores of oesophageal cancer patients were compared to those of a reference population of randomly selected Swedish inhabitants matched by age and gender to a previously used population-based cohort of Swedish oesophageal cancer patients [20]. In addition, adjusted comparisons of 1 year HRQOL scores were made between different subgroups within the curative and palliative subcohorts described above.

HRQOL data were prospectively measured at 1 year after diagnosis within the NREV framework. These questionnaires were sent by mail to the patients that were still alive and after completion returned to the registry data center to be registered in the NREV database.

All items in the EORTC HRQOL questionnaires were included in the analyses. The EORTC core questionnaire (QLQ-C30) contains nine multi-item scales, measuring global quality of life, functions (physical, role, emotional, cognitive and social) and multi-item symptom scales (fatigue, nausea and vomiting, and pain), and six single items measuring general cancer symptoms (dyspnoea, sleeping problems, loss of appetite, constipation, diarrhoea and financial problems) [17]. The disease specific oesophageal module questionnaire (QLQ-OG25) is divided into one function scale measuring body image and six-multi item symptom scales (dysphagia, eating, reflux, odynophagia, pain, discomfort and anxiety) and nine single item scales (eating with others, dry mouth, trouble with taste, trouble swallowing saliva, choked when swallowing, trouble with coughing, weight loss and hair loss) [18]. However, the item hair loss is only answered if this symptom has been experienced. All items use a 4-point Likert scale from [1] “not at all”, [2] “a little”, [3] “quite a bit”, and [4] “very much”, except for the global health/quality of life scale having a seven-point scale ranging from [1] “very poor” to [7] “excellent”.

#### Statistical analysis

Data from all items were linearly transformed on a scale between 0 to 100, and missing data were handled according the manual from the questionnaire developers [21]. A high score on the functional scales or the global quality of life scale indicates a high function or high level of global quality of life, conversely a high score on a symptom scale represents a high level (severity or frequency, depending on the specific question) of the symptom in question. To aid interpretation for clinical mean differences (MD), within the EORTC QLQ-C30 instrument, we used evidence-based guidelines on cross-sectional data [22]. Based on previous research for the oesophageal module EORTC QLQ-OG25 and emotional function in QLQ-C30, a difference of ≥10 in mean score between comparison groups was considered clinically relevant [23, 24], and a difference of ≥20 mean in score was considered a large difference. Moreover, a *p*-value less than 0.05 was considered significant in all statistical analyses. All comparisons regarding mean scores in function or symptom scales were evaluated with regard to clinical relevance, which was considered a mandatory prerequisite for statistical significance testing. Linear regression was used to calculate mean scores and mean score differences (MD) with 95% confidence intervals (CI). All MD analyses were adjusted for the following potential confounding factors: age (as a continuous variable), gender (binary categorical variable), histology (multilevel categorical variable) and for the curative intent cohort also T-stage (multilevel categorical variable), but excluding T-stage in T0-T1 subcohort.

All analyses were performed with the statistical software Stata® 14.2 (Stata Corporation, College Station, Texas, USA).

#### Ethics

The study was approved by the regional ethics review board of Stockholm county (Dnr. 2013/ 596–31/3 and 2016/1486–32). Patient consent was waived, as the study is based on pseudonymised registry-data.

## Results

### Baseline characteristics of patients still alive 1 year after diagnosis

**Fig. 1:**
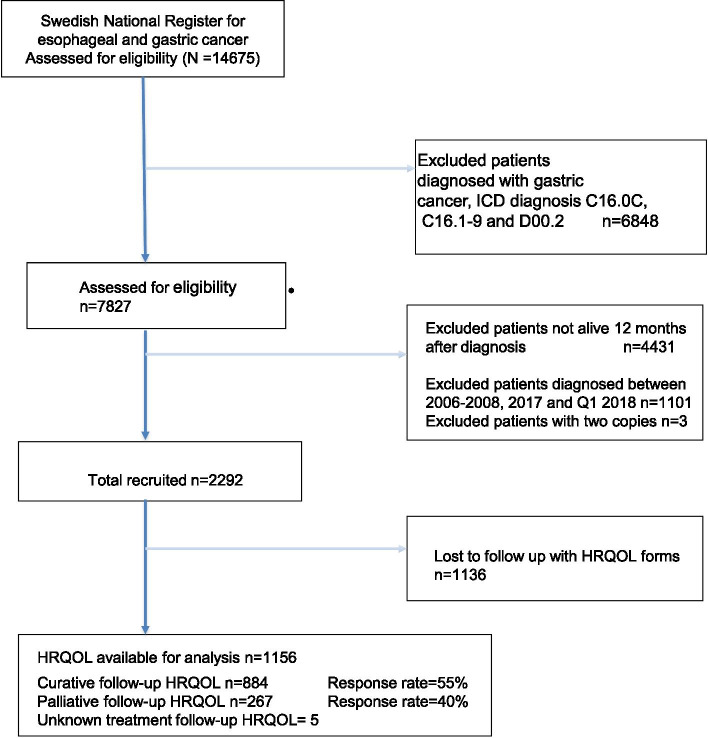
STROBE flowchart of included patients who completed the EORTC QLQ-C30 and QLQ-OG25

A total of 14,675 individuals with oesophageal, gastric or GOJ cancers, were registered, in NREV between January 2006 and May 2018 Among these 7827 individuals were diagnosed with oesophageal or GOJ Siewert type I and II cancers, of which 2292 patients met the full inclusion criteria of being diagnosed with their cancer between January 2009 and December 2016 and were alive 1 year after the date of diagnosis. Of these 2292 patients 1156 responded (50.4%) to the HRQOL questionnaires and were included in the analyses of HRQOL 1 year after diagnosis (Fig. 1).

HRQOL questionnaires were completed by more than 55% of the curative intent patients, while only 40% of those with a palliative management intention responded (Table 1, Fig. 1). No differences were detected between the HRQOL questionnaire responders and non-responders, regarding baseline characteristics including age, gender, performance status (WHO), ASA comorbidity score, histological subtype and clinical stage (Table 1). Palliative intent patients generally had worse performance status and more advanced clinical stage disease, especially regarding M-stage, compared to curative intent patients (Table 1).

**Table 1:** Characteristics of all patients alive 1 year after oesophageal cancer diagnosis

	Curative intent		Palliative intent	
	HRQOL^a^ data		HRQOL^a^ data	
	Yes	No	Yes	No
**Responders:**				
**Total**	884	709	267	411
**Sex ratio (M: F)**	683:201	559:150	193:74	299:112
**Age (years)**	66 (29–93)	65 (20–89)	72 (37–91)	70 (21–95)
**WHO performance status**	867	689	260	398
**0**	493 (57)	406 (59)	69 (26)	124 (31)
**1**	327 (38)	212 (31)	121 (47)	153 (38)
**2**	44 (5)	65 (9)	56 (22)	87 (22)
**3**	3 (0)	6 (1)	13 (5)	32 (8)
**4**	0 (0)	0 (0)	1 (0)	2 (0)
**Missing**	17	20	7	13
**ASA comorbidity score**	871	697	259	400
**1**	297 (34)	236 (34)	49 (19)	75 (19)
**2**	443 (51)	338 (49)	107 (41)	163 (40)
**3**	126 (14)	114 (16)	86 (33)	143 (36)
**4**	5 (1)	9 (1)	17 (7)	19 (5)
**Missing**	13	12	8	11
**Tumor location**				
**Proximal**	53 (6)	45 (6)	14 (6)	33 (8)
**Middle**	81 (9)	78 (11)	25 (11)	45 (11)
**Distal**	643 (73)	502 (71)	169 (73)	237 (58)
**Not specified**	107 (12)	82 (12)	24 (10)	95 (23)
**Histological type**	878	701	265	410
**Adenocarcinom**	614 (70)	464 (66)	175 (66)	260 (63)
**SCC**^**b**^	191 (22)	175 (25)	59 (22)	102 (25)
**Other**	73 (8)	62 (9)	31 (12)	48 (12)
**Clinical T stage**	884	708	267	410
**T0-T1**	145 (16)	132 (19)	40 (15)	46 (11)
**T2–3**	595 (67)	466 (65)	138 (52)	224 (55)
**T4**	36 (4)	54 (8)	38 (14)	69 (17)
**Tx**	108 (12)	56 (8)	51 (19)	71 (17)
**Clinical N stage**	884	708	267	410
**N0**	529 (60)	382 (54)	104 (39)	136 (33)
**> N1**	322 (36)	291 (41)	134 (50)	213 (52)
**Nx**	33 (4)	35 (5)	29 (11)	61 (15)
**Clinical M stage**	883	707	267	406
**M0**	851 (96)	673 (95)	155 (58)	214 (53)
**M1**	16 (2)	19 (3)	110 (41)	181 (44)
**Mx**	16 (2)	15 (2)	2 (1)	11 (3)

^a^Health-related quality of life and ^b^Squamous cell carcinoma

Comparing those who completed European Organisation for Research and Treatment of Cancer their general questionnaire QLQ-C30 and oesophageal module QLQ-OG25 and those who did not. Percentage within each category in brackets

### HRQOL 1 year after diagnosis in all patients and compared to the reference population

#### General cancer HRQOL instrument EORTC QLQ-C30

Mean scores were lower for several functions among palliative compared to curative intent patients (Global health/QOL, physical function, role function, emotional function and social function). Functions and global health/QOL were in general lower in both the curative and palliative cohort compared to the reference population. A much higher symptom burden was reported among oesophageal cancer patients, compared to the reference population, regarding all symptoms investigated (Table 2).

**Table 2:** Health-Related Quality of Life at 1 year follow-up in oesophageal and GOJ cancer in patients

	All* n = 1156	Reference population ***n*** = 4910	Curative treatment ***n*** = 884	Palliative treatment ***n*** = 267
**QLQ-C30**	Mean (CI)	Mean (CI)	Mean (CI)	Mean (CI)
Global health/QOL	60 (58 to 62)	76 (76 to 77**)**	61 (60 to 63)	55 (51 to 58)
**Functions**				
Physical function	73 (72 to 75)	88 (87 to 89)	76 (75 to 78)	64 (61 to 68)
Role function	64 (62 to 66)	88 (88 to 89)	66 (64 to 68)	56 (51 to 60)
Emotional function	75 (74 to 76)	86 (85 to 86)	76 (75 to 78)	71 (67 to 74)
Cognitive function	82 (80 to 83)	88 (88 to 89)	82 (81 to 84)	79 (76 to 82)
Social function	71 (69 to 72)	91 (91 to 92)	72 (70 to 74)	65 (61 to 69)
**Symptoms**				
Fatigue	43 (41 to 44)	19 (18 to 20)	41 (39 to 42)	49 (46 to 53)
Nausea and vomiting	17 (15 to 18)	3 (2 to 3)	16 (15 to 18)	18 (15 to 21)
Pain	26 (25 to 28)	19 (18 to 20)	25 (23 to 27)	31 (27 to 35)
Dyspnoea	36 (34 to 38)	16 (16 to 17)	35 (33 to 37)	41 (37 to 45)
Insomnia	28 (26 to 30)	18 (17 to 18)	27 (25 to 29)	31 (27 to 35)
Loss of appetite	30 (28 to 32)	3 (3 to 4)	28 (26 to 30)	38 (33 to 43)
Constipation	14 (12 to 15)	5 (5 to 6)	11 (10 to 13)	22 (18 to 25)
Diarrhoea	21 (19 to 22)	6 (5 to 6)	23 (21 to 25)	13 (10 to 16)
Financial	14 (12 to 16)	4 (4 to 5)	14 (13 to 16)	13 (10 to 16)
**QLQ-OG25**				
**Function**				
Body Image	72 (70 to 74)		74 (71 to 76)	67 (63 to 72)
**Symptoms**				
Dysphagia	24 (22 to 26)		22 (20 to 24)	32 (28 to 36)
Eating	33 (32 to 35)		32 (30 to 34)	37 (33 to 41)
Reflux	24 (22 to 25)		24 (23 to 26)	21 (18 to 25)
Odynophagia	20 (18 to 21)		19 (17 to 20)	23 (20 to 27)
Pain and discomfort	26 (25 to 28)		26 (24 to 28)	27 (23 to 31)
Anxiety	46 (44 to 48)		44 (42 to 46)	52 (48 to 56)
Eating with others	21 (19 to 22)		18 (16 to 20)	29 (25 to 34)
Dry mouth	30 (28 to 32)		29 (26 to 31)	36 (32 to 40)
Trouble with taste	24 (22 to 26)		22 (20 to 24)	31 (27 to 36)
Trouble swallowing saliva	12 (11 to 14)		11 (10 to 13)	17 (13 to 20)
Choked when swallowing	18 (16 to 19)		17 (15 to 19)	20 (16 to 23)
Trouble with coughing	31 (29 to 32)		30 (28 to 32)	31 (27 to 35)
Trouble talking	12 (10 to 13)		11 (10 to 13)	14 (11 to 17)
Weight loss	31 (29 to 33)		30 (28 to 33)	33 (29 to 38)
Experience of Hair loss *(fewer responders)*	21 (18 to 24)		22 (18 to 25)	19 (13 to 25)

* All patients answering questionnaire, including palliative, curative and five patients with unknown treatment intention

Mean scores and 95% confidence intervals (CI) in patients who completed European Organisation for Research and Treatment of Cancer QLQ-C30 questionnaire and oesophageal module QLQ-OG25, describing all patients and stratified by curative and palliative treatment intent, and also including a Swedish reference population

#### Oesophageal specific HRQOL instrument QLQ-OG25

The mean function score for body image, especially among those with palliative management intention, was very low. Both curative and palliative intent patients reported severe symptoms of problems eating, anxiety, dry mouth, cough and weight loss, while palliative patients also reported problems with dysphagia and taste (Table 2).

### HRQOL 1 year after diagnosis in the curative intent patients, stratified by T-stage and curative treatment type

#### General cancer HRQOL instrument EORTC QLQ-C30

Among patients treated with curative intent there were no clinically relevant differences detected in the comparison of function scores between T0-T1 patients and T2-T4 patients that were operated and T2-T4 patients treated with dCRT. For this reason no statistical significance testing was performed (Table 3).

**Table 3:** Health-Related Quality of Life at 1 year follow-up including curative intent oesophageal and GOJ cancer patients

	cT0-T1	cT2–4 Surgery	cT2–4 dCRT	cT0-T1 compared to cT2–4 Surgery adjusted	cT0-T1 compared to cT2–4 dCRT adjusted	cT2–4 Surgery compared to cT2–4 dCRT adjusted
QLQ-C30	Mean (CI) **n (145)**	Mean (CI) **n (539)**	Mean (CI) **n (92)**	MD (CI)	MD (CI)	MD (CI)
Global health/QOL	64 (60 to 69)	61 (59 to 63)	58 (53 to 64)	−4 (−9 to 1)	− 6 (−13 to 2)	− 2 (−8 to 4)
Functions						
Physical function	79 (76 to 82)	76 (74 to 78)	72 (67 to 77)	−3 (−8 to 1)	−6 (−12 to 0)	−4 (−9 to 1)
Role function	71 (66 to 76)	66 (63 to 69)	65 (59 to 71)	−5 (−11 to 2)	− 5 (− 14 to 4)	−1 (−9 to 7)
Emotional function	76 (72 to 80)	76 (75 to 79)	75 (70 to 79)	0 (− 5 to 5)	−3 (− 10 to 3)	−2 (− 7 to 3)
Cognitive function	83 (79 to 86)	83 (81 to 85)	79 (74 to 84)	0 (−4 to 4)	−5 (−11 to 1)	− 4 (− 9 to 0)
Social function	76 (71 to 80)	72 (70 to 74)	70 (64 to 76)	−4 (−9 to 2)	− 7 (− 14 to 1)	−3 (− 9 to 4)
Symptoms						
Fatigue	35 (31 to 40)	41 (39 to 44)	45 (39 to 50)	6 (1 to 12)	9 (1 to 16)	3 (−3 to 9)
Nausea and vomiting	11 (8 to 15)	18 (16 to 20)	13 (9 to 18)	5 (0 to 9)	0 (−6 to 5)	−5 (−10 to 0)
Pain	27 (22 to 31)	25 (22 to 27)	25 (19 to 30)	−3 (−8 to 3)	−2 (−9 to 5)	2 (− 4 to 8)
Dyspnoea	31 (27 to 36)	35 (32 to 38)	36 (30 to 42)	5 (−1 to 11)	6 (−2 to 14)	1 (−6 to 8)
Insomnia	24 (19 to 29)	29 (27 to 32)	23 (17 to 28)	3 (−3 to 9)	−2 (− 10 to 6)	−6 (− 13 to 1)
Loss of appetite	20 (15 to 25)	29 (26 to 32)	30 (22 to 37)	10 (3 to 16)	7 (−2 to 16)	0 (−8 to 8)
Constipation	12 (8 to 16)	11 (9 to 13)	14 (9 to 19)	−2 (−6 to 3)	1 (− 6 to 8)	3 (− 2 to 8)
Diarrhoea	21 (16 to 26)	26 (23 to 28)	11 (7 to 15)	3 (−3 to 8)	**−10 (− 17 to − 3)**	**−14 (− 20 to − 8)**
Financial	11 (7 to 16)	15 (13 to 17)	14 (8 to 19)	1 (−4 to 6)	2 (−5 to 9)	1 (− 5 to 7)
QLQ-OG25						
Function						
Body Image	79 (74 to 84)	73 (70 to 76)	70 (63 to 78)	−7 (−13 to 0)	−9 (− 18 to 0)	−1 (− 9 to 7)
Symptoms						
Dysphagia	18 (13 to 23)	22 (19 to 24)	34 (27 to 42)	5 (−1 to 11)	**16 (8 to 25)**	**11 (4 to 18)**
Eating	24 (20 to 28)	34 (32 to 36)	34 (28 to 40)	**10 (5 to 15)**	8 (1 to 16)	−1 (−7 to 5)
Reflux	23 (18 to 28)	25 (23 to 28)	20 (14 to 25)	2 (−3 to 8)	−4 (−12 to 3)	−8 (− 14 to − 1)
Odynophagia	18 (13 to 22)	18 (16 to 20)	23 (18 to 29)	−1 (−6 to 4)	3 (−4 to 10)	5 (0 to 11)
Pain and discomfort	26 (22 to 31)	27 (25 to 29)	23 (17 to 28)	0 (−5 to 5)	−5 (−12 to 3)	−4 (− 10 to 2)
Anxiety	40 (35 to 44)	44 (42 to 47)	47 (39 to 54)	4 (−2 to 10)	7 (−1 to 16)	3 (−4 to 10)
Eating with others	15 (10 to 20)	18 (16 to 21)	23 (17 to 29)	4 (−1 to 10)	9 (1 to 17)	4 (−3 to 11)
Dry mouth	27 (22 to 32)	28 (26 to 32)	28 (21 to 35)	3 (−3 to 9)	2 (−6 to 11)	−1 (−9 to 6)
Trouble with taste	18 (13 to 22)	22 (19 to 25)	28 (21 to 35)	4 (−2 to 10)	9 (1 to 18)	6 (−1 to 13)
Trouble swallowingsali**v**a	8 (5 to 11)	12 (10 to 14)	14 (9 to 19)	3 (−2 to 7)	5 (−1 to 11)	2 (−4 to 7)
Choked when swallowing	14 (11 to 18)	16 (14 to 18)	27 (20 to 35)	3 (−2 to 8)	**13 (5 to 20)**	**10 (4 to 16)**
Trouble with coughing	28 (23 to 32)	31 (29 to 34)	32 (25 to 39)	3 (−3 to 9)	1 (−7 to 10)	0 (−7 to 7)
Trouble talking	9 (6 to 13)	11 (9 to 13)	19 (12 to 25)	5 (0 to 9)	**13 (6 to 20)**	5 (−1 to 10)
Weight loss	19 (14 to 24)	34 (31 to 37)	27 (20 to 34)	**18 (11 to 25)**	9 (1 to 17)	−8 (−16 to 0)
Hair loss	23 (12 to 36)	21 (17 to 26)	23 (11 to 36)	−5 (−18 to 9)	−9 (−28 to 10)	−3 (− 15 to 10)

Values in bold are both clinically relevant and statistically significant

Mean scores and adjusted mean score differences (MD) with 95% confidence intervals (CI) in patients who completed the European Organisation for Research and Treatment of Cancer QLQ-C30 questionnaire and the oesophageal module QLQ-OG25, stratified by clinical T-stage

Regarding the symptom panel, high scores were reported regarding fatigue and dyspnoea, both for T0-T1 and T2-T4 patients. There was a clinically relevant difference, which was also statistically significant, with more diarrhoea (MD -14; 95% CI − 20 to − 8) in T2-T4 patients that were operated compared to those treated with dCRT and also between T0-T1 patients and those T2-T4 patients treated with dCRT (MD-10, 95% CI − 17 to − 3) (Table 3).

#### Oesophageal specific HRQOL instrument QLQ-OG25

High mean scores for anxiety were reported in all categories of patients initially managed with curative intent. Also, high scores were reported regarding problems with coughing in all curative patient categories, but no clinically relevant differences were observed in comparisons between the subgroups.

Patients with T2-T4 tumours treated with dCRT reported more problems with dysphagia, than those operated (MD 11; 95% CI 4 to 18) and compared to those with T0-T1 tumours (MD 16, 95% CI 8 to 25). Patients with T2-T4 tumours that were treated with resectional surgery reported more trouble with eating (MD 10, 95% CI 5 to 15) and more weight loss (MD 18, 95% CI 11 to 25) than T0-T1 patients. In addition T2-T4 patients treated with dCRT choked more when swallowing, both compared to T0-T1 patients (MD 13, 95% CI 5 to 20) and compared to T2-T4 patients that were operated (MD 10; 95% CI 4 to 16). Lastly, patients with T2-T4 tumours treated with dCRT reported more problems talking than those with T0-T1 tumours (MD 13, 95% CI 6 to 20). These differences were all of clinically relevant magnitude and statistically significant (Table 3).

### HRQOL 1 year after diagnosis in palliative intent patients, stratified by the reason for palliative management

#### General cancer HRQOL instrument EORTC QLQ-C30

The mean global health/qol was scored low in all palliative groups, irrespective of the reason for palliative intent management. Also, functions (physical and role) are reported with low mean scores in all subgroups. When comparing the subcohorts there were no differences of clinical relevance in general functions assessed in the QLQ-C30 questionnaire (Table 4).

**Table 4:**
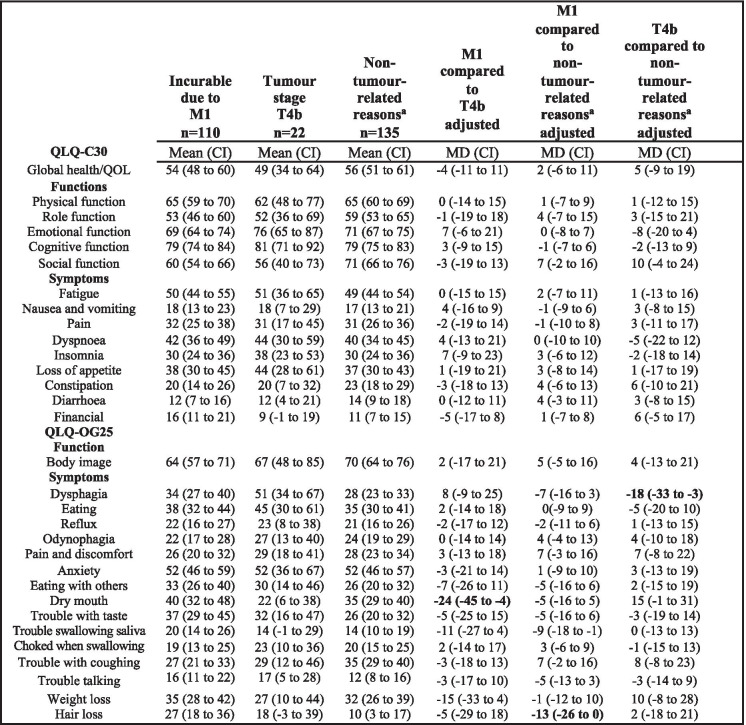
Health-Related Quality of Life at 1 year follow-up in palliative intent oesophageal and GOJ cancer patients

Values in bold are both clinically relevant and statistically significant

Mean scores and adjusted mean score differences (MD) with 95% confidence intervals (CI) in patients who completed the European Organisation for Research and Treatment of Cancer QLQ-C30 questionnaire and oesophageal module QLQ-OG25, stratified by reason for palliative intent

*Abbreviations*: *M1* Palliative intent due to distant metastatic disease, *T4b* Palliative intent due to tumour invasion of adjacent organs or structures

^a^Patients with palliative intent due to frailty and/or comorbidities

Regarding the symptoms assessed in the QLQ-C30 general cancer questionnaire, there were no clinically relevant differences between the palliative intent subgroups (Table 4).

#### Oesophageal specific HRQOL instrument QLQ-OG25

Symptoms assessed in the oesophago-gastric cancer specific instrument QLQ-OG25 differed regarding some symptoms, between the studied palliative subcohorts. Patients with palliative management intent due to distant metastasis had more problems with dry mouth, to a clinically relevant extent, than those with palliative intent due to locally advanced, irresectable primary tumours (MD -24; 95% CI − 45 to − 4) (Table 4). Patients with palliative management due to distant metastasis reported more problems with hair loss, compared to those with palliative treatment intent due to non tumour-related factors (MD -13; 95% CI − 26 to 0) (Table 4), to a clinically relevant extent. Patients with palliative management because of locally irresectable tumour had more dysphagia than patients classified as palliative due to non-tumour-related reasons (MD -18; 95% CI − 33 to − 3) to a clinically relevant degree. All the above described clinically relevant differences were also statistically significant (Table 4).

## Discussion

In this population-based cohort study of HRQOL 1 year after diagnosis of oesophageal or GOJ cancer, overall low function scores and high symptom scores were reported, especially in comparison with an age and gender matched reference population. As expected, patients managed with a palliative treatment intention up-front had generally lower function scores and higher symptom burden than those initially managed with a curative intention. Within the curative intent subcohort, patients after surgery reported more diarrhoea than those treated with dCRT, while the latter group reported more problems with eating, dysphagia and choking while eating. Within the palliative intent subcohort, patients classified as incurable due to locally advanced and irresectable tumours reported more dysphagia than those managed palliatively for non-tumour related reasons. Patients deemed incurable due to distant metastatic disease reported more problems with dry mouth compared to those with locally advanced primary tumour. Patients with distant metastatic disease also reported more problems with hair loss than those with palliative intent due to locally advanced tumour.

Some methodological issues need to be considered. A strength of the study is the prospective, population-based data collection, which counteracts selection and recall bias. Compared to randomised controlled trials population-based data may better reflect the unselected clinical reality, better representing the whole patient population and therefore possibly adding value to health care policy and decision-making. Another strength of the study is that NREV has been validated and found to have very high validity, reliability and coverage [19] and also the HRQOL questionnaires used have been formally validated [17, 18]. By analysing all HRQOL items and scores, there is a risk of chance findings, but we consider this risk has been minimised by only performing statistical significance testing of differences of clinically relevant magnitude [22–24].

There are also some limitations that need to be considered. Missing data is a weakness in this cohort study, some regional centres did not collect PROM during the first years of HRQOL data collection. Missing data is a well-known problem when collecting PROM, especially in randomised clinical trials with poor outcomes [3, 25]. National cohort studies collecting HRQOL from patients with oesophageal cancer, are hithertho uncommon, but in our view essential in order to better understand patients’ functions and symptoms within the different clinical pathways [6, 16]. The definition of the subcohorts are based on only one time-point shortly after diagnosis and staging and it is likely that some of the patients have not been treated according to the intention registered, and others are likely to have had a change of decision and treatment intention at some later point in time. This is especially likely for initially curatively managed patients who within the first year may have had a recurrence and then changed to palliative management intention.

One year after diagnosis, patients suffering from cancer have gone through personal changes with time, treatment experiences and disease progression [8, 9, 26]. This adoption process is described as response-shift, a change in personal values (reprioritization), internal standards (recalibration) or meaning in definition of HRQOL (reconceptualization) [26]. This was taken into consideration when using the evidence based guidelines in the interpretation of clinical relevance, consequently these response-shift effects are likely to be similar in all subgroups.

Problems with diarrhoea were more pronounced in the advanced T-stage surgical subcohort, and also in the T0-T1 subcohort, compared with patients with locally advanced tumours treated with dCRT. This is known from previous literature and most likely caused by the surgery, probably to a large extent influenced by the vagotomy usually performed as an inherent part of the dissection [10, 27, 28]. Currently T0-T1 tumours are mostly offered only endoscopic treatment, while in the early years of this data collection a large proportion were treated with oesophagectomy. In contrast, patients treated with dCRT had more dysphagia and eating-related problems than those operated. A well known side-effect of radiotherapy is radiation-induced oesophagititis, which together with the long-term radiotherapy effect of fibrosis and stricture, may cause the reported swallowing and eating symptoms in patients after dCRT. It’s also likely that the high prevalence of dysphagia in the dCRT sub-group may be influenced by selection of more locally advanced tumours not considered to be radically resectable.

The findings of this study suggest that in patients with oesophageal cancer in general, but in particular those with a palliative management intention, attention needs to be focused on alleviating psychological distress, anxiety, and on treating dysphagia and other eating related problems, as well as recognition of altered physical and social roles. With emerging new treatments, such as immunotherapy, survivorship is likely to increase, which further stresses the importance of treating symptoms and incrementally working on improvement of quality of life.

## Conclusions

In conclusion, in this poplation-based cohort study unselected patients diagnosed with oesophageal cancer and alive 1 year after diagnosis reported generally low HRQOL function scores and high HRQOL symtom scores compared to an age and gender adjusted reference population. Dysphagia, choking, and other eating related problems were more pronounced in palliatively managed patients and in those curatively treated with dCRT, compared to those that were surgically resected. These findings strongly suggest that individualised management of symptoms and support of mental and physical functions is still an unmet need in the care of patients with oesophageal cancer, both in the palliative and curative settings.

## Data Availability

The dataset used and analysed during the current study is available from the corresponding author on reasonable request.

## Abbreviations

ASA: American Society of Anesthesiologists comorbidity score
CI: Confidence intervals
dCRT: Definitive chemoradiotherapy
EORTC: European Organisation for Research and Treatment of Cancer
GOJ: Gastro-oesophageal junctional
HRQOL: Health related quality of life
PROM: Patient reported outcome measures
M1: Distant metastatic disease
MD: Mean differences
MDT: Multi-disciplinary conference
NREV: Swedish National Registry for Oesophageal and Gastric Cancer
T0-T4b: Tumour stages
EORTC QLQ-C30: European Organisation for Research and Treatment of Cancer Core Questionnaire
EORTC QLQ-OG25: European Organisation for Research and Treatment of Cancer disease specific Questionnaire for oesophageal and gastric cancer
WHO: Performance status
